# Barriers to Using Mobile App-Based Cognitive Testing in Older Adults with Probable Alzheimer’s Disease: A Qualitative Study

**DOI:** 10.3390/brainsci15050464

**Published:** 2025-04-27

**Authors:** Mei-Lan Chen, Kun-Lin Hsieh, Sung-Lin Hsieh, Ming-Cheng Hsieh, Dan Chia-Tien Lo, Jung-Lung Hsu

**Affiliations:** 1 School of Nursing, Byrdine F. Lewis College of Nursing and Health Professions, Georgia State University, Atlanta, GA 30303, USA; 2 School of Computer Science, Georgia Institute of Technology, Atlanta, GA 30332, USA; khsieh37@gatech.edu; 3 Department of Computer Science, School of Engineering, Vanderbilt University, Nashville, TN 37235, USA; sung-lin.hsieh@vanderbilt.edu; 4 North America Taiwanese Engineering and Science Association, Sunnyvale, CA 94086, USA; mhsieh@natea.org; 5 Department of Computer Science, Kennesaw State University, Marietta, GA 30060, USA; dlo2@kennesaw.edu; 6 Department of Neurology, New Taipei Municipal TuCheng Hospital (Built and Operated by Chang Gung Medical Foundation), New Taipei City 236, Taiwan; 7 Graduate Institute of Mind, Brain and Consciousness, Taipei Medical University, Taipei 110, Taiwan

**Keywords:** dementia, Alzheimer’s disease, mobile app-based cognitive assessment, older adults, qualitative study, family caregivers, thematic analysis, smart technology, digital cognitive testing, cognitive function

## Abstract

**Background/Objectives**: Smart technologies have the potential to be rapid, sensitive, and cost-effective tools for real-life monitoring of cognitive function among older adults. Identifying barriers to using remote cognitive testing in older adults’ daily lives is essential. However, obstacles of utilizing digital cognitive tests in community-dwelling older adults with probable Alzheimer’s Disease (AD) remain unclear. The purpose of this study was to investigate barriers of older adults with probable AD toward using mobile app-based cognitive assessment. **Methods**: This was a qualitative study. Older adults with probable AD and their family caregivers were recruited from an outpatient clinic at a hospital’s neurological department. Semi-structured interviews were performed. Characteristics of the participants were analyzed using descriptive statistics. Thematic analysis was conducted to identify barriers to using mobile app-based cognitive testing. **Results**: The study sample consisted of 32 older adults with probable AD and 19 family caregivers involving the use of at-home mobile app-based cognitive assessment every two weeks over 12 months. In this study, three main themes were identified, including barriers related to availability of family support, health-related barriers, and app-related barriers. **Conclusions**: Mobile app-based cognitive assessment could be a promising tool for detecting AD and monitoring cognition among older adults. A better understanding of the barriers to using at-home digital cognitive testing can facilitate the development of patient-centered and custom-built mobile applications for older adults with probable AD and improve their cognitive function.

## 1. Introduction

Dementia has become a prominent issue for older adults, negatively impacting individuals, families, communities, healthcare systems, and societies [1,2,3,4]. More than 55 million people in the world have dementia [1]. That number is expected to double every 20 years, increasing to 78 million by 2030 and 139 million by 2050 [5]. The annual cost of dementia is more than $1.3 trillion worldwide, and that number is predicted to increase to $2.8 trillion by 2030 [5]. Globally, dementia is the seventh leading cause of death and a significant cause of disability and dependency among older adults [1,6]. Therefore, promoting older adults’ cognitive health to reduce the burden of dementia is essential.

Alzheimer’s Disease (AD), the most common type of dementia, is a progressive and fairly disabling neurodegenerative disease [7,8]. Through observation of dominantly inherited AD, global cognitive impairment can be detected several years before clinical diagnosis and then dramatically decreased as the disease advanced [7,9,10,11]. However, there exists a high rate of unrecognized cognitive impairments among older adults [12]. Previous research revealed that only 41% of older adults with probable dementia were diagnosed and had awareness of dementia, leaving older adults with undiagnosed dementia at high health risks [12,13]. Hence, real-life monitoring and early detection of cognitive impairment among community-dwelling older adults will be essential for mitigating the significant challenges that these older adults and their family caregivers face [12,14,15].

Standard in-clinic cognitive assessments are expensive and time-consuming [16]. Among cognitively healthy older adults, compared with traditional in-clinic testing methods, mobile app-based cognitive assessments have shown to be more feasible and reliable [17]. For detecting AD-related cognitive impairments in older adults, mobile app-based cognitive assessments have demonstrated to be more feasible, reliable, and valid than traditional in-clinic testing methods [16,18,19]. Overall, previous studies have shown that mobile app-based cognitive assessments can be a reliable, cost-effective, and rapid tool for continuously monitoring the cognitive health of older adults [20,21,22].

Even with the promising potential of mobile app-based cognitive assessments in monitoring the cognitive health of older adults, many older adults face obstacles to using technologies [23,24]. Previous studies have identified possible barriers to using technologies among older adults, such as costs, lack of knowledge and information, negative attitudes, age-related challenges (e.g., declines in vision, hearing, and motor functions), technology-related anxiety and resistance, level of technology experience, platform management and infrastructure, and privacy issues [23,24,25,26]. A better understanding of users’ needs and experience can enhance the development of custom-built smart technology and reduce obstacles to digital technology usage [24,26]. However, few studies have focused on the experience of older adults with probable AD toward using technologies. Barriers to using at-home digital cognitive assessments among older adults with probable AD remain unclear. Accordingly, the purpose of this qualitative study was to explore barriers of older adults with probable AD to utilizing mobile app-based cognitive assessment for real-time monitoring of cognitive function.

## 2. Materials and Methods

### 2.1. Ethical Approval

This study was approved by the Institutional Review Board of Chang Gung Memorial Hospital in Taiwan (CGMHIRB No. 202201446B0).

### 2.2. Study Design

This was a qualitative study. Older adults with probable AD and their family caregivers were recruited from an outpatient clinic at a hospital’s neurological department in Taiwan. Each patient completed the dementia diagnosis based on cognitive evaluations, brain image study, and other laboratory studies before this one. Written informed consent was obtained from each subject before the study procedure.

In the current study, SymptomTrace App, an at-home digital cognitive assessment application, was developed for both Android and iOS system smartphones to allow remote self-administration for tracking cognition. In brief, the digital cognitive assessments included picture description task, picture naming task, pentagon drawing task, and category verbal fluency task [27,28,29,30]. Each task was briefly described as follows:

(a) Picture description task: The subject saw one of three sets of Taiwanese pictures and was asked to describe the scene depicted. Subjects were given the following voice instructions: “Describe everything you see on this picture card.” There was no time limit for this task [27].
(b) Picture naming task: For each assessment, subjects perceived 3 pictures and were asked to name the objects. They had up to 20 s to name each item [28].
(c) Pentagon drawing task: Subjects were instructed to copy or draw two interlocking pentagons, with the interlocking shape being a diamond [29].
(d) Category verbal fluency task: One of the four categories (i.e., animals, fruit, vegetables, fish) was used to test subjects’ verbal fluency at each time point. Subjects were asked to name as many items as possible in each of these categories within 60 s [30].

Participants were asked to complete the at-home digital cognitive assessments using their own smartphones via the SymptomTrace app every two weeks for one year (a total of 24 sessions). Each participant received an account and QR code to build their database on the cloud platform. Subjects’ information was stored using SHA256 encryption to ensure data security. Participation in this study was completely voluntary. Participants had the right to drop out at any stage of the study without any penalty.

### 2.3. Participants

Patients aged between 55 and 75 years old, with education of more than 6 years, are fluent in Mandarin Chinese, and with mild to moderate severity of dementia were eligible to participate in this study. The severity of dementia was defined by Mini-Mental Status Examination (MMSE) 10–26 scores or Clinical Dementia Rating (CDR) 0.5–2 scores [29,31]. The clinical diagnosis of probable AD was based on the National Institute on Aging and Alzheimer’s Association (NIA-AA) criteria [32]. Subjects with stroke, brain tumor or other organic brain diseases, psychiatric disorders (e.g., anxiety, major depressive disorder), severe visual or hearing impairment, or communication problems were excluded. Patients and their caregivers who have never used a smartphone were excluded from the study.

### 2.4. Data Collection

A self-reported questionnaire was used to collect participants’ demographic information and research-related data. Following the 12-month SymptomTrace app use, acceptability measure and semi-structured interviews were completed. Acceptability of the SymptomTrace app was quantified by the following measures: (a) perceived usefulness of the app; (b) liked/disliked; (c) ease of use; (d) satisfaction; (e) willingness in continuing to use the app in the future. Acceptability measures were rated on a 5-point scale with five Likert-type responses. Higher scores indicate higher acceptability and satisfaction.

Semi-structured interviews via phone were conducted to obtain qualitative data for gaining an in-depth understanding of barriers of older adults with probable AD to utilizing the SymptomTrace app for digital cognitive assessments [33]. All interviews were directed by an interview guide with open-ended questions, probes, and prompts [34,35]. Interview topics included “experiences, perceptions, and satisfaction with the app use”, “advantages and disadvantages of the use of the app”, “any barriers when using the app”, “suggestions for the app design”, and “willingness in continuing to use the app in the future” [36]. All interviews were audio recorded, transcribed verbatim, and anonymized.

### 2.5. Data Analysis

Participants’ demographic characteristics and acceptability of the SymptomTrace app were analyzed using descriptive statistics. Statistical analyses were performed using Statistical Package for the Social Sciences (SPSS, version 29.0, IBM, Armonk, NY, USA).

Braun and Clarke’s six-phase thematic analysis method was used for qualitative data analysis [37,38,39]. In Phase 1, data familiarization was completed by reading and re-reading all transcripts multiple times and taking notes for coding. Initial codes were generated from the entire data set in Phase 2. For searching for themes (Phase 3), the codes were categorized into potential themes. During Phases 4 and 5, each theme was reviewed, defined, refined, and named. Lastly, in Phase 6, the final report for the results of this thematic analysis was written.

Trustworthiness and rigor of the study were ensured through regular analysis meetings, debating and defending sessions, and investigator triangulation [40]. To solve any disagreements or conflicts, the investigators participated in discussions until a consensus was reached.

## 3. Results

### 3.1. Characteristics of the Sample

A total of 51 participants (32 older adults with probable AD and 19 family caregivers) were included in this study. All participants completed this qualitative study. The mean completion time of cognitive testing was 17.4 ± 6.1 sessions. Patients with probable AD ranged in age from 64 to 75 (mean age 70.5 years ±2.5); caregivers ranged in age from 37 to 76 (mean age 56.5 years ±12.9). Of the total sample, 18 patients and 8 caregivers self-reported as female. In terms of living arrangement, 96.9% of patients lived with family. Details on the characteristics of the participants are shown in Table 1.

### 3.2. Acceptability of the SymptomTrace App for At-Home Digital Cognitive Assessment

Full details on the acceptability of the SymptomTrace app are indicated in Table 2. In brief, 73.1% of participants found that the SymptomTrace app was “useful/very useful” and 80.8% were willing to continue use the app in the future. About 61.6% of participants liked the design of the app and 65.4% were satisfied with using the app. Overall, most participants (80.8%) reported that it was “easy/very easy” to use the app.

### 3.3. Barriers to Using the SymptomTrace App for At-Home Digital Cognitive Assessment

A total of 32 semi-structured interviews were conducted. Three main themes emerged from qualitative data using thematic analysis, including barriers related to availability of family support, health-related barriers, and app-related barriers. The three main themes and representative quotes for each main theme are presented in the following sections.

#### 3.3.1. Theme 1: Barriers Related to Availability of Family Support

The first theme identified from qualitative data was barriers related to availability of family support. Most patients reported that they needed their family members’ help and reminders to use the app for at-home digital cognitive testing. They indicated that without the help from their family members, it was difficult for them to use the app. Some caregivers also emphasized that the patients are not able to use the app if their family members are not at home to help and/or remind them.

*“Sometimes my family members are not available. It will be difficult for me to use this app, if my family members cannot assist in using the app”.*

(Patient 13)

*“If my family members don’t remind me, I will forget to use the app”.*

(Patient 25)

*“My children were not with me, so I often forgot to take the test”.*

(Patient 34)

*“I live alone. It is not easy for me to use the app, because I do not have the support from my family members to help me to use the app”.*

(Patient 33)

*“Without the help of family members, it will be difficult for the patient to use this app”.*

(Caregiver 2)

*“The patient always forgot to use the app. He needed to have family members’ assistance in using the app”.*

(Caregiver 10)

*“If family members are not at home, the patient will not use the app”.*

(Caregiver 11)

*“If family members are not there to help and remind the patient, he is not able to take the test”.*

(Caregiver 26)

#### 3.3.2. Theme 2: Health-Related Barriers

The second theme identified from qualitative data was health-related barriers. Participants reported health problems as the barrier to the use of the app for cognitive assessments, such as memory and emotional problems.

*“Due to my physical health problem, taking the test is a frustration for me”.*

(Patient 8)

*“I am old and have health problems. My response becomes slower. I am confused about the content of the question. The test is too difficult for me”.*

(Patient 13)

*“I have memory problems. When looking at pictures that tell stories, it’s hard to understand the situation the pictures are trying to express”.*

(Patient 32)

*“It was difficult for me to use this app, due to health reasons”.*

(Patient 3)

*“For the patient, the questions are difficult to understand. She has memory problems. She does not know what the questions are about. The patient felt stressed when taking the test”.*

(Caregiver 2)

*“The patient has emotional problems. She felt depressed and frustrated when taking the test. She has memory problems; the test was difficult for her”.*

(Caregiver 8)

*“I feel that the patient’s health conditions and personality have become lazy, so he does not want to take the test”.*

(Caregiver 26)

#### 3.3.3. Theme 3: App-Related Barriers

The final theme identified from qualitative data was app-related barriers. Many patients indicated that they seldom use their smartphones. Participants reported app-related problems as the barrier to utilizing the app for cognitive testing, such as user interface and operation issues, font size, and login problems.

*“It was not easy for me to use the app. The app crashed, and the system was unstable”*.

(Patient 10)

*“I rarely use the smartphone. The pictures are too small, and I cannot see clearly. I cannot see clearly even if I wear glasses. The text is too small. I cannot see clearly even if I wear glasses”*.

(Patient 8)

*“The reminder function is too few and it is easy to ignore or forget to use the app”*.

(Patient 14)

*“I seldom use my mobile phone. The operation is not smooth, and I frequently get logged out”*.

(Patient 34)

*“I rarely use my smartphone. It was very common to be unable to log in, preventing me from taking the test. The font size has become smaller, and I did not know how to adjust it”*.

(Patient 24)

*“It is difficult for me to use the app. The font is too small. The user interface is not intuitive”*.

(Caregiver 3)

*“It was not easy for the patient to take the test. The image size cannot be zoomed in. The pictures are difficult to see clearly”*.

(Caregiver 15)

*“The app did not save progress when it crashed. If you have to ask the patient to do it again, the patient will be unwilling to do it again”*.

(Caregiver 6)

*“It was difficult for the patient to use the app for the test. For example, in the drawing part, if the grid is too small, the patient will draw outside the grid and has to keep redrawing, which will make the patient impatient”*.

(Caregiver 17)

## 4. Discussion

Little is known about the barriers to using digital cognitive assessments among older adults with probable AD. To fill this gap, this qualitative study aimed to investigate barriers of older adults with probable AD to utilizing app-based, at-home cognitive testing for real-time monitoring of cognition. In this study, semi-structured interviews were conducted to gain an in-depth understanding of the experiences and perspectives of older adults with probable AD and their caregivers regarding the barriers to the use of SymptomTrace app for digital cognitive assessments. The strength of the current study is that, in addition to the perspective of the older adults with probable AD for one-year experience of the app use, it also includes the perspective of the family caregivers. Three main themes yielded by using thematic analysis were barriers related to the availability of family support, health-related barriers, and app-related barriers.

In line with previous research [41,42,43,44], the result of this study revealed that many older adults with probable AD seldom use smartphones in everyday life. Compared with young adults, older adults are less likely to engage actively with digital technologies [41,42,43,45]. This may be due to the fact that some older adults with low educational attainment reside in rural areas and do not own or have access to technologies [46]. Also, educational limitations can cause limited levels of language and digital literacy, preventing older adults from effectively using technologies [47]. In addition, income and technology prices may greatly impact older adults’ access to digital technologies [47]. Thus, older adults’ socioeconomic status may determine their abilities to access and utilize digital technologies. For future research, the role of social determinants in promoting digital equity and inclusion for older adults with probable AD should be considered.

In this study, barriers related to the availability of family support have been identified as one of the key themes that older adults with probable AD face toward using mobile app-based cognitive assessments. Previous studies have supported that a lack of family support can contribute to the barriers against older adults to using digital technologies [23,41,48,49]. Family members who are not available or not willing to help can act as a barrier to older adults’ digital technology usage, literacy, and skills, particularly in older adults with lower socioeconomic status [41,48]. Also, the willingness of older adults to receiving technology-related support from their family is significantly associated with the perception of the support possibilities and the family members’ willingness to support older adults [49,50]. Hence, for reducing barriers to the acquisition of digital skills and literacy with smartphones among older adults with probable AD, it is essential to enhance and strengthen family support, especially intergenerational relationships and interactions.

Consistent with previous research [23,46,47], findings from the current study indicated health-related barriers (e.g., emotional problems, cognitive impairments) as another main theme for the utilization of mobile app-based cognitive assessments among older adults with probable AD. The existing literature suggest hearing and vision impairments, as well as fine motor difficulties, as barriers of older adults toward using digital technologies [23]. This is not surprising because vision problems can negatively impact the ability of older adults to read and navigate digital systems and apps [23,46]. Cognitive impairments can also reduce the ability of older adults to understand and learn instructions on using digital technologies [23,47]. In addition, physical limitations can cause pain, numbness, and shakiness in the hands, causing difficulties in using smartphones [47]. Moreover, older adults may experience technology-related anxieties, fears, and resistance toward using technologies [24]. Some older adults have fears, anxieties, and mistrust while navigating through apps and the Internet due to their technology-related concerns about electric shocks, safety, and privacy threats [47,51,52]. As health-related barriers can impact the ability of older adults with probable AD to use app-based cognitive testing, it is necessary to modify digital technology in terms of physical, mental, and cognitive challenges related to the disease and digital skills.

The present study identified app-related barriers (e.g., the perceived difficulties of use, small font size) as one of main themes for barriers to using mobile app-based cognitive testing in older adults with probable AD. Similarly, the existing literature indicated that an app’s touch screens, font sizes and colors, button sizes, and logging in issues can affect the use of digital systems and apps among older adults [47,52]. Prior studies also showed that the perception of difficulties in using apps, user interface design issues, difficult setup, and confusing instructions are barriers to the utilization of smart technology [51,52]. For future research, simple, readable age-friendly apps that can be personalized and contain educational resources regarding challenges related to digital technologies should be developed for older adults with probable AD and their family caregivers [52,53].

As for mitigating the barriers of older adults with probable AD toward using mobile app-based cognitive assessments, the current study suggests that it is imperative to build support networks with their family and friends in helping them use the apps, as well as providing emotional support to lessen technology-related anxiety [41,49,50,54]. In addition, developing age-friendly, reliable, and secure applications and devices that are tailored to the needs, health conditions, and preferences of each older user is essential [24,55]. It is very important to take into consideration creating a personalized digital design with interactive features, as well as the deployment of a simplified and patient-centered user interface to reduce users’ physical, cognitive, and emotional burden [24,26,55]. Moreover, a potential way to mitigating the barriers could be to execute technological training sessions for older adults to help them adapt to and become familiar with using digital technologies, especially for older adults who live alone without any family members [24,50,54]. Personalized training and education sessions that are tailored to the needs, conditions, and requirements of each individual older adult are highly recommended [52,54]. For future research and clinical practice, to overcome barriers of older adults with probable AD toward using digital health technologies, providing the users with more encouragement, support, and collaborative and cognitively stimulating learning environments and activities are essential [41,50,52,54,56].

This study has several potential limitations. Patients ranged in age from 64 to 75 years. Adults older than this age range were not involved in this study. Moreover, many patients reported that they seldom use smartphones. Also, this study did not assess patients’ digital literacy. It remains unclear whether patients who regularly use smartphones or have higher digital literacy levels also face the same barriers toward using the app for at-home digital cognitive testing. In addition, most patients reported living with family members. All patients are community-dwelling older adults who were recruited from an outpatient clinic in Taiwan. It may have limited the transferability of the study findings.

## 5. Conclusions

The utilization of smart technology may potentially detect AD and monitor cognitive functioning in older adults. This qualitative study provides a better understanding of the barriers to using mobile app-based cognitive assessment in daily life among older adults with probable AD. Three main themes were identified using thematic analysis, including barriers related to availability of family support, health-related barriers, and app-related barriers. For future research and clinical practice, personal, social, physical, and mental contexts should be considered when designing mobile applications for older adults with probable AD to overcome the barriers toward the use of mobile app-based cognitive assessment.

## Figures and Tables

**Table 1:** Characteristics of the sample (*N* = 51).

Variable	Patient (*n* = 32)	Caregiver (*n* = 19)
Age (years)	70.5 ± 2.5	56.5 ± 12.9
(mean ± SD)		
Sex [*n* (%)]		
Female	18 (56.3%)	8 (42.1%)
Male	14 (43.7%)	11 (57.9%)
Education (years) (mean ± SD)	10.0 ± 3.7	13.0 ± 2.6
Time Since Diagnosis of Probable AD (years; mean ± SD)	4.4 ± 1.7	
Patient’s Living Arrangement [*n* (%)]		
Live Alone	1 (3.1%)	
Live with Family Member(s)	31 (96.9%)	
Caregiver’s Relationship to Patient [*n* (%)]		
Spouse		9 (47.4%)
Daughter		5 (26.3%)
Son		5 (26.3%)

Note: SD = Standard Deviation; AD = Alzheimer’s Disease.

**Table 2:** Acceptability of the SymptomTrace app for at-home digital cognitive assessment.

Item	Rating	
	*n* (%)	Mean ± SD ^a^
App was Useful		3.8 ± 0.5
Agree/Strongly agree	19 (73.1%)	
Neither agree nor disagree	6 (23.1%)	
Disagree	1 (3.8%)	
Liked the Design of the App		3.6 ± 0.6
Agree/Strongly agree	16 (61.6%)	
Neither agree nor disagree	9 (34.6%)	
Disagree	1 (3.8%)	
App Ease of Use		3.8 ± 0.5
Agree/Strongly agree	21 (80.8%)	
Neither agree nor disagree	3 (11.5%)	
Disagree	2 (7.7%)	
Satisfied Using the App		3.7 ± 0.6
Agree/Strongly agree	17 (65.4%)	
Neither agree nor disagree	8 (30.8%)	
Disagree	1 (3.8%)	
Willingness to Continuing to Use the App in the Future		3.9 ± 0.6
Agree/Strongly agree	21 (80.8%)	
Neither agree nor disagree	2 (7.7%)	
Disagree	3 (11.5%)	

Note: ^a^ All items scored as 1 = strongly disagree, 2 = disagree, 3 = neither agree nor disagree, 4 = agree, 5 = strongly agree; SD = Standard Deviation.

## Data Availability

The data are not publicly available due to privacy and ethical restrictions.
